# Pathophysiology of vascular dementia

**DOI:** 10.1186/1742-4933-6-13

**Published:** 2009-11-06

**Authors:** Francesco Iemolo, Giovanni Duro, Claudia Rizzo, Laura Castiglia, Vladimir Hachinski, Calogero Caruso

**Affiliations:** 1 Department of Clinical Neurological Sciences University of Western Ontario, Ontario, Canada; 2 Robarts Research Institute Stroke Prevention & Atherosclerosis Research Centre, London, England, UK; 3 U.O. di Neurologia, Ospedale di Vittoria, ASL7-Ragusa and Clinica Neurologica Università di Catania, Catania, Italia; 4 Istituto di Biomedicina ed Immunologia Molecolare-Consiglio Nazionale delle Ricerche (IBIM-CNR), Palermo, Italy; 5 U.O. di Immunoematologia e Medicina Trasfusionale, AOUP "Paolo Giaccone", Palermo, Italy; 6 Dipartimento di Biopatologia e Metodologie Biomediche, Università di Palermo, Palermo, Italy

## Abstract

The concept of Vascular Dementia (VaD) has been recognized for over a century, but its definition and diagnostic criteria remain unclear.

Conventional definitions identify the patients too late, miss subjects with cognitive impairment short of dementia, and emphasize consequences rather than causes, the true bases for treatment and prevention. We should throw out current diagnostic categories and describe cognitive impairment clinically and according to commonly agreed instruments that document the demographic data in a standardized manner and undertake a systematic effort to identify the underlying aetiology in each case.

Increased effort should be targeted towards the concept of and criteria for Vascular Cognitive Impairment and Post-Stroke Dementia as well as for genetic factors involved, especially as these categories hold promise for early prevention and treatment.

## Background

The concept of Vascular Dementia (VaD), has been recognized for over a century, but its definition and diagnostic criteria remain unclear and generate, much confusion and debate althoung several clinical criteria have been used for defining the VaD

The term of VaD substantially means "disease with a cognitive impairment resulting from cerebrovascular disease and ischemic or hemorrhagic brain injury".

Dementia represents only a portion of the burden of cognitive dysfunction associated with cerebrovascular disease. In addition to patients who develop dementia, there are those who develop cognitive impairment that does not fulfill traditional criteria for dementia but that nonetheless has a significant impact on quality of life and ability to carry out activities of daily living. As a result, the older term "vascular dementia" is being replaced with a new one: "vascular cognitive impairment" (VCI). In addition, postmortem pathological studies indicate that 15% to 34% of dementia cases show significant vascular pathology, either alone or in combination with Alzheimer disease (AD) pathology [1].

In fact, dementia criteria are typically modelled on Alzheimer Disease (AD), wherein involvement of the mesial temporal lobe results in dense episodic memory impairment. [2,3]. Current research has led to the identification of a cognitive syndrome signalling a high risk of further cognitive decline, a group of patients similar to that of mild cognitive impairment as a risk state for AD [4]. The cognitive impairment attributable to cerebrovascular disease is a rapidly escalating public health problem. The heterogeneity of the population of patients with VaD diagnosed using current criteria has raised the need for updating the classification of subtypes

### History

The concept of "dementia" originated early in the 16^th ^century., when for the first time Pratensis mentioned the "Dementia stroke correlate". in the "De cerebri morbis -1549" Subsequently Willis described the most important causes of dementia including ageing and vascular disease and the first accurate clinical observations of patients with vascular dementia In the early 19^th ^century Cooke described "intellectual deficits among the sequelae of apoplexy" The history of vascular dementia during 19^th ^and 20^th ^centuries has been recently reviewed [5].

Binswanger and Alzheimer, began a series of clinicopathological correlation studies attempting to isolate additional forms of dementia, describing four different form of vascular dementia (arteriosclerotic brain degeneration, perivascular gliosis of the brain cortex, dementia post-apoplexian and Binswanger's chronic progressive subcortical encephalitis) [6-16]. Pierre Marie described in 1901 the "***état lacunaire***" associated with a constant intellectual deficits. Dementia was separated from delirium and other psychiatric diseases at the end of the 19^th ^century, but its etiology remained un cleared [17].

Alzheimer and Binswanger had correctly concluded that "arteriosclerotic dementia" represented a large clinico-pathological spectrum. However, "arteriosclerotic dementia" incorrectly became synonymous with senile dementia, and it was widely held that cortical atrophy in the elderly resulted from progressive decrease in cerebral perfusion leading to hypoxic neuronal death.

Little further progress was made for the next 70 years because of a combination of factors (Freud's work, Kraepelin's classification, syphilis as a common and obscuring cause of dementia). New suggestions and observations were made, but they did not thrive against this background. In 1946 a clear description of a multi-infarct dementia was given, recognizing the possible role of silent infarcts and explicating that the chronic ischemia wasn't he cause of vascular dementia, but the true mechanism was the infarction. This process later termed "multi-infarct dementia" [18]

Today, the importance of vascular lesions in Alzheimer's disease is being increasingly recognized: various data shows that more than 30% of AD exhibit cerebrovascular pathology [5].

A recent review define the AD as a vascular disorder: "...since the value of scientific evidence generally revolves around probability and chance, it is concluded that the data presented here pose a powerful argument in support of the proposal that AD should be classified as a vascular disorder. According to elementary statistics, the probability or chance that all these findings are due to an indirect pathological effect or to coincidental circumstances related to the disease process of AD seems highly unlikely. The collective data presented in this review strongly support the concept that sporadic AD is a vascular disorder." [19].

## Discussion

### Vascular dementia (VAD) and vascular cognitive impairment (VCI): clinical criteria

Cardinal elements implemented in the clinical criteria for VaD are the definition of the cognitive syndrome of dementia and the objective documentation of vascular lesions capable of causing dementia [20-24]. All the currently clinical criteria, are derived from expert opinion based on prevailing knowledge and pathogenetic hypothesis of dementia's causes [25].

The current clinical criteria for VaD recognize the multiplicity of lesions, but none of these clinical criteria provide guidelines for subtype of VaD [26]. Only the NINDS-AIREN criteria mention the following subtype: cortical vascular dementia, Binswanger's disease and thalamic dementia [27]. Also the brain imaging requirements are not included, or are not complete, in all current clinical criteria.

The systematic heterogeneity of the population of patients with VaD diagnosed by using current criteria has raised the need for updating the classification of subtypes. The main subtype of VaD include: multi-infarct dementia or predominantly cortical VaD, strategic infarct dementia and small vessel dementia or subcortical VaD even if only subcortical VaD is probably a more homogenous group [28-31]

The new criteria are based on *homogeneity *(in aetiologies, brain imaging and clinical syndrome), on *predictability *(phenomenology and clinical picture, clinical course and natural history, outcomes and treatment responses), on *reproducibility *(intra- and inter-rater reliability). Furthermore, they must be validated by prospective clinical pathological correlation [25,32].

The cornerstone of all criteria of dementia is memory impairment; this criterion works very well for AD, in which the mesial temporal lobes are affected early and prominently with consequent early memory loss, but strokes affecting cognition occur most commonly in the frontobasal systems that subserves judgement, planning and emotion, features seldom tested in cognitive screens.

At present, there is no generally accepted test battery for identifying or classifying patients with VCI. However, there are some basic principles that can be followed in developing such a battery. One of these is that large vessel cortical strokes and subcortical small vessel disease, tend to produce different kinds of deficits. The former typically present with region-specific syndromes such as aphasia, apraxia, and amnesia. The latter present with more subtle and temporally progressive deficits, often described as "executive" in nature. [4,33-37]. These include deficits in speed and so-called "strategic" processing (ie, attention, planning, and monitoring) in tasks such as memory tasks. Patients may perform normally on simple tasks but reveal deficits as tasks increase in complexity. It appears that the majority of VCI patients fall into the class with subcortical small vessel disease. [21,38]. Thus, it seems reasonable that neuropsychological testing for VCI would include tasks testing executive function. In addition, such tests may help to differentiate patients in which either vascular or AD pathologies predominate [1]

The extent of ischaemic disease on neuroimaging, that is both sufficient and necessary to cause cognitive impairment, Leukoaraiosis and atrophy as well as the issue of location [39]. To summarize the literature data, it's clear that there are insufficient data to propose firm cut-offs for the extent of Leukoaraiosis or for extent of infarction.

Anyway, enough scientific rationale already exists for undertaking systematic clinical trials in the prevention of cognitive impairment through the control of vascular risk factors and the use of statins, anti-inflammatory agents, ACE inhibitors, vitamins E and B12 [40].

### Epidemiology

Epidemiologic studies of VaD have been hampered by the "lack of clear and universal diagnostic criteria, by the use of different strategies in detecting dementia cases of vascular origin, by the difficulties of developing an effective case-finding strategy, and by the complexity of using imaging or laboratory tests in large scale epidemiologic surveys" [41]. Despite these difficulties, the broad contours of VaD epidemiology are emerging slowly [42-44].

The data from current studies cannot be compared and reconciled easily. Diagnostic criteria for VaD have been a long standing source of disagreement and are perhaps the greatest barrier to reaching consensus on the epidemiology of this disease [42,43,45]. A first problem is the classification of patients with the so-called "mixed" dementia, in whom the aetiology appears to include both cerebrovascular and primary degenerative features. A second problem is the disagreement about criteria and evaluation of tools. A number of new sets of criteria have been introduced in the 1990s, including the ICD-10, the Chui et al., the Roman et al., and the DSM-IV criteria [46,47]. An additional problem with epidemiologic studies is the need to transfer published criteria into clinical instruments or procedures that can be implemented on a large scale. Brief scales to separate VaD from AD have been developed beginning with the Hachinski Ischemic Score (HIS) introduced in 1975. A number of modifications or transformations of this original scale have been proposed in the past 20 years [48]. Some of these scales require imaging tests, whereas others are purely clinical. A third problem is the use of imaging findings in the definition of VaD: in the incidence study of dementia in Rochester, the age-specific incidence rate of VaD by using more restrictive DSM-IV criteria (clinical stroke required) and less restrictive criteria (certain vascular imaging lesion sufficient) diverge beyond 70 years of age, and are more than doubled with the restrictive criteria beyond 85 years of age [49]. A fourth problem is to define the range of severity of VaD to be detected in the study: severity may vary from a mild cognitive impairment to terminal stages of deterioration.

If the prevalence of VaD is greater among women than men of a certain age, it remains uncertain whether women have a higher incidence of VaD or whether the incidence is equal to men, but survival after onset of VaD is better in women [41].

Today new prevalence studies are available) and the breadth of variation for VaD prevalence is now even wider. It remains unclear whether the differences between different countries reflect disagreement over diagnostic criteria for VaD or other methodological differences. For studies investigating the incidence of VaD in different countries the data are similarly disparate [50,34].

The data available to answer the question if the prevalence or the incidence of VaD is increasing or decreasing over time are very few. Because the data from current studies cannot be easy reconciled, it remains very difficult to draw conclusions about the epidemiology of VaD. Our progress would benefit greatly from the development of a new set of diagnostic criteria sensitive and specific, easy to apply in the field setting and internationally accepted. It appears, however, that the prevalence of VaD tends to be higher in men than in women, and that it increases with age; moreover there seems to have been a decline in both prevalence and incidence of VaD between the 1950s and 1970s [41]. The prevalence of VaD ranged typically between 3% and 6%; but the variation described ranged between 0% to 20%. There are relatively few data regarding incidence in the general population. The incidence of cognitive impairment sufficient to adversely affect outcome but not meeting current criteria for vascular dementia is as high as 35.2% compared to 3.8% with a similar degree of impairment in stroke-free controls [18,35,42].

### Pathophysiology

Epidemiological studies have explored widely the potential risk factors or protective factors for different dementing disorders [36,51]. Many studies have focused on AD and more recently, on all types of dementia as a syndrome. Aetiological studies about VaD have been hampered by methodological issues and - the search for risk factors of VaD may potentially be more amenable to prevention than AD [52].

Some common determinants between AD and VaD are:

- risk factors involved in cerebrovascular disease (age, sex, some atherogenic disorders or vascular risk factors, genetic factors and inflammation). Other potential risk factors like occupational exposure to pesticide, psychological stress or life events, dietary fat intake, family history of stroke, etc.);

- potential protective factors (high educational attainment, eating fish or shellfish, physical exercise, use of supplementary antioxidants like Vitamins E and C, use of Vitamin B12, Mediterranean diet, etc.) [36,50,53].

Vascular cognitive impairment is not a regular pathogenetic entity. Multiple small thromboembolic strokes or strokes in strategic locations such as the thalamus, frontal lobe or temporal lobes may cause cognitive impairment and frequently occur without classical stroke-like symptoms. Nonetheless, in VaD, the majority of patients instead present widespread microangiopathy-related cerebral damage, which is often clinically silent or is accompanied by unspecific neurological signs.

Several mechanisms may explain why patients affected by stroke are prone to develop dementia [20,37,54,55]:

- post-stroke dementia may be the direct consequence of vascular lesions in the brain

- post-stroke dementia could be the result of pre-existing neuropathological effects AD's related

- recurrent stroke that is cause by vessel damages and by white matter lesions that may lead to cognitive decline and contribute to post-stroke dementia;

One of the mechanism involved in ischemic VaD is under the control of large vessels disease (atherosclerosis, and other arteriopathies), however, impaired cerebral flow in the absence of infarct as consequence of arterial stenosis has been documented, although its clinical consequences remain to be fully investigated. It is also unclear whether and to what degree large vessel disease contributes to the white matter pathology and lacunes associated with the subcortical type of VaD. Statistical association suggests it may have additive effects to small vessel pathology. [56,57]

Moreover, the alterations of small vessels play a role in causing damage to the cerebral tissue and are potentially responsible for the subsequent development of cognitive alterations. Small vessel lesions are considered related to the deep lacunar infarcts and white matter changes typically observed in subcortical forms of vascular cognitive impairment.

The most common types of diseases affecting cerebral microvessels are: arteriosclerosis, lipohyalinosis, cerebral amyloid angiopathy, basal ganglia calcification, CADASIL (Cerebral Autosomal Dominant Arteriopathy with Subcortical Infarcts and Leucoencephalopathy), other uncommon intracerebral vasculopathies.

The small vessel alterations could also lead to damage affecting the blood-brain barrier and chronic leakage of fluid and macromolecules in the white matter, even if the neuroimaging methods do not detect diffuse alterations of the blood-brain barrier in vivo [58].

Another mechanism influencing small vessel alterations could be incomplete ischemia and selective tissue necrosis (i.e. incomplete infarction) causing a selective neuronal necrosis with sparing of glial cells and microvessels [59-62].

Recently, it has been suggested a new variety of lacunar infarction, (type Ib), characterized by small areas of perivascular rarefaction and selective neuronal loss, followed in the more advanced stages by a varying amounts of depleted neurons and oligodendrocytes, astroglial response and minor central cavitation. Furthermore, embolic occlusion of small penetrating artery followed by spontaneous lyses of the thrombus could be responsible for these lacunes [63-65].

The concept of Leukoaraiosis, suggested by Hachinski, was introduced to describe morphological abnormalities of the white matter on imaging [66].

The pathogenesis of white matter changes (WMC) is not well established and a number of possible mechanisms have been hypothesized, all mechanisms are reconducible to a form of cerebrovascular disease. It has been proposed that the diffuse changes of the white matter should be considered a form of incomplete infarction. [67-76]

The understanding of the pathobiology of AD and VaD recived an impulse by the discovery of genes that produce monogenic forms of the illness or contribute to polygenic forms; in particular, the identification of genes contributing to VCI would no doubt provide insight into the cellular and molecular basis of VCI.

Genetic factors play an important role in the aetiology of VaD, in particular, it's seems to be more important in large-vessel stroke and small vessel stroke than in cryptogenic stroke, and there is no epidemiological evidence for a genetic component in cardioembolic stroke.

The genes underlying VaD must be of 2 nonmutually exclusive classes: *(1) *genes that predispose individuals to cerebrovascular disease, and *(2) *genes that determine tissue responses to cerebrovascular disease (eg, genes conveying ischemic tolerance or susceptibility, or the ability to recover from ischemic insult). With regard to the first class of genes, some progress has been made in the past few years in identifying genes that confer susceptibility to hypertension and stroke [77]. In addition, several monogenic forms of cerebrovascular disease have been identified. The two best studied of these are cerebral autosomal dominant arteriopathy with subcortical infarcts and leucoencephalopathy (CADASIL: a subcortical small vessel disease accompanied by lacunar strokes, migraine, and dementia) and hereditary cerebral hemorrhage with amyloidosis-Dutch type (HCHWA-D) [1].

The CADASIL condition is a heritable small-vessel disease caused by mutations in NOTCH3 gene which is normally expressed in vascular smooth muscle cells and pericytes (including those of the cerebral vasculature) and that encodes a cell-surface receptor, which has a role in arterial development and is expressed on vascular smooth-muscle cells. appears to be involved in directing smooth muscle cell proliferation and differentiation. The NOTCH3 receptor is a heterodimer composed of a large extracellular fragment and a smaller transmembrane intracellular fragment. About 95% of patients have missense mutations that cluster in exons 3- and consist in change of cysteine residues amount, but the pathogenic meccanism is still unknown [78,79].

Whit regard to HCHWA-D (a syndrome of primarily hemorrhagic strokes and dementia), it is caused by a mutation in the gene for amyloid precursor protein (APP) that causes abnormal deposition of amyloid in the walls of leptomeningeal arteries and cortical arterioles (a mpathological condition known as cerebral amyloid angiopathy [CAA]). Mouse models have been developed for CADASIL and HCHWA-D and have contributed critical insights into the cell biology of the pathogenic processes underlying them. [80]

In contrast, little attention has been paid to the second class of genes: those that render the brain more or less susceptible to injury in response to cerebrovascular disease. Evidence for the existence of such response genes is that patients with apparently similar loads of vascular pathology (with regard to lesion type, number, and location) may range from no cognitive impairment to severely cognitively impaired. The association studies using the candidate gene approach, has identified a number of genetic variants possibly involved in risk factor development and, on the other hand, suggests that the risk of stroke has a substantial genetic component. Nonetheless, the genes underlying this risk in the general population remain undetermined. Genetic factors can act at several levels. They can contribute to conventional risk factors such as hypertension, diabetes, or homocysteine concentrations, which have a known genetic components. They might further interact with environmental factors or contribute directly to an intermediate phenotype [78,81]

It is also possible that some genetic factors contribute, by interaction with conventional risk factors, to the development of subcortical injury of vascular origin in non familiar cases.

Most single-gene disorders are associated with specific stroke subtypes, especially in young stroke patients without known risk factors. Table 1; ref. [79,82-87].

**Table 1:** Single-gene disorders associated with ischaemic stroke (for reference see the text)

*Gene*	*Disease*	*Mode of * *inheritance*	*Stroke mechanism*
***GAL***	*Fabry's disease*	*X-linked*	*Large-artery disease and small-vessel disease*
***NOTCH3***	*CADASIL*	*AD*	*Small-vassel disease*
***HBB***	*Sickle-cell disease*	*AR*	*Large-artery disease, small-vessel disease, haemodynamic insufficiency*
***CBS and others***	*Homocystinuria*	*AR*	*Large-artery disease, cardioembolism, small-vessel disease, arterial dissection*
***mtDNA***	*MELAS*	*Maternal*	*Complex (microvascular and neuronal factors)*
***FBN1***	*Marfan syndrome*	*AD*	*Cardioembolism and arterial dissection*
***COL3A1***	*Ehlers-Danlos syndrome type IV*	*AD*	*Arterial dissection*
***ABCC6***	*Pseudoxanthoma elasticum*	*AR*	*Large-artery disease and small-vessel disease*

AD = autosomal dominant. AR = autosomal recessive. HBB = haemoglobin beta. CADASIL = cerebral autosomal dominant arteriopathy with subcortical infarcts and leucoencephalopathy. MELAS = mitochondrial myopathy, encephalopathy, lactacidosis, and stroke. mtDNA = mitochondrial DNA.

For references, see text

In this group of diseases we take in consideration only Fabry's disease for its special association with stroke. The Fabry's disease is an X-linked systemic disorder caused by deficiency of the lysosomal enzyme α-galactosidase A, that results in progressive accumulation of glycosphingolipids in the myocardium, renal epithelium, skin, eye, and vasculature. The symptoms are acroparesthesia, angiokeratoma, hypoidrosis, which start during the childhood or adolescence and are present in the majority of the affected individuals and many young patients are affect by cryptogenetic stroke. The disease occurs either in large-artery either in small-vessel disease, with a preference for posterior circulation. Small-vessel disease in Fabry's disease is associated with white-matter changes and evidence suggests that the extent of such lesions is influenced by gene polymorphisms as interleukin 6, endothelial nitric oxide synthase, factor V, and protein Z [78,82]. These findings are suggestive but need further confirmation (Duro's data not published).

The genetic contribution to common multifactorial stroke seems to be polygenic: there are many alleles with small effect, due to their wide distribution, however, on a population basis the impact on stroke is large. Several genes and polymorphisms were selected for a significant association with ischaemic stroke: in particular polymorphisms in the genes encoding MTHFR (enzyme in homocysteine metabolism), ACE (enzyme in renin-angiotensin-aldosterone system), Factor V Leiden, prothrombin and PAI 1 (Haemostatic system) [78]

Recently, studies for the analyses of ischemic stroke assessed two unsuspected common SNPs on chromosome 12p13 (rs11833579 and rs12425791), consistently associated with total ischemic, and atherothrombotic stroke in caucasic population although there is association with ischemic and atherothrombotic strokes (as compared with total stroke), but no association with non ischemic stroke. The SNPs are on the gene NINJ2 that encodes an adhesion molecule expressed in glia cells and shows increased expression after nerve injury Some mutations are associated with a slightly more aggressive phenotype. [81].

One class of genes that must influence tissue responses to cerebrovascular disease are the AD genes. There is an additive or synergistic interaction between AD and cerebrovascular pathologies, such that individuals with both of these pathologies show greater cognitive impairment than those exhibiting either pathology alone. In addition, at least three sets of genes in the AD pathway, the presenilins, APP, and apolipoprotein E (apoE), are known to interact with the VCI disease pathway.

The presenilins, mutations of which cause AD, have been shown to interact directly with Notch proteins, including Notch 3 (mutations of which cause CADASIL).

Mutations in the APP gene can lead either to AD or to hemorrhagic stroke and dementia (as in HCHWA-D) depending on the site of the mutation and the subsequent cellular site of amyloid accumulation.

Variants of the apoE gene appear to affect not only susceptibility to cerebrovascular disease but also recuperative responses to it (see below). Thus, there appear to be links in the biochemical pathways underlying VCI and AD pathologies, which could be responsible for the observed interactive effects of these pathologies on cognitive function.

Genes that influence brain responses to cerebrovascular disease do not appear to be limited to those within AD pathway. First, it has been shown that VCI can occur in the complete absence of AD pathology in sporadic VCI and in hereditary forms. In addition, the cognitive sequelae of pathogenic processes associated with VCI are different from those seen in "pure" AD, in that executive function appears more strongly affected in VCI than is memory. Consistent with these observations, different brain regions seem differentially affected in VCI and AD, with prefrontal circuits being more affected in VCI and the hippocampus in AD.

An important aspect of VaD's (AD's particular) pathophysiology, is the role of inflammation: the incidence is influenced by the gene polymorphisms of the inflammatory mediators. Among the most widely investigated genes are those involved in inflammation (interleukin 1, interleukin 6, TNFα, toll-like receptor 4, P-selectin and E-selectin, C-reactive protein), lipid metabolism (apolipoprotein E, paraoxonase, epoxide hydrolase), nitric oxide release, and extracellular matrix (matrix metalloproteinases) [88,89].

Infact, in recent years, an increasing set of evidence has stress the role of inflammation in the brain, particularly in the microglia-rich amyloid deposits, where the microglias tend to release a wide variety of proinflammatory mediators including cytokines (IL 1β, IL 6, TNF α, acute phase proteins) complement components, various free radicals and nitric oxide (NO), all of which potentially contribute to further neuronal dysfunction and eventually result in cellular death. In addition, apolipoprotein E (ApoE) is strongly associated with AD in terms of cognitive decline and disease onset: ApoE, especially the ε4 allele, has been observed promoting an inflammatory reaction[14]. The AD's pathomechanism is related to the accumulation of toxic amyloid-β (Aβ), which precipitate along the vessel walls and in brain parenchymal plaques, as well as the formation of neurofibrillary tangles (NFTs); the development of cognitive impairment in VaD may be related to a number of different pathological processes including multiple infarctions resulting from occlusion of major brain vessels or their branches and lypohyalinosis. The latter is a degeneration of small arterial vessel walls supplying the subcortical white matter, thalamus, and basal ganglia, or cerebral amyloid angiopathy (CAA is the accumulation of amyloid protein in the walls of cerebral blood vessels): less commonly, vascular dementia is related to deposition of Aβ in brain vessels. CAA can be either sporadic or familial. The progressive cognitive impairment in CAA results from multiple brain hemorrhages and/or ischemia related to narrowing of vessel lumen [88,90]

## Conclusion

We should throw out current diagnostic categories and describe cognitive impairment clinically and according to commonly agreed instruments that document the demographic data in a standardized manner and undertake a systematic effort to identify the underlying aetiology in each case (imaging and DNA should be obtained whenever possible.

However, further empirical research and international debate is needed to define the syndrome and stages of vascular subcortical cognitive impairment, validate the brain imaging criteria for subcortical Vascular Dementia by clinical-pathological correlation, as well as the natural history and outcomes of the syndrome.

Increased effort should be targeted towards the concept of and criteria for Vascular Cognitive Impairment and Post-Stroke Dementia as well as for genetic factors involved, especially as these categories hold promise for early prevention and treatment.

## Competing interests

The authors declare that they have no competing interests.

## Authors' contributions

All authors drafted, read and approved the final manuscript.
